# Identification and validation of a novel apoptosis-related prognostic risk score model for lung adenocarcinoma

**DOI:** 10.7150/jca.92616

**Published:** 2024-04-28

**Authors:** Yan Wang, Jiaojiao Zhang, Yong Wan, Baibing Mi, Manxiang Li, Xinming Xie

**Affiliations:** 1 Department of Respiratory and Critical Care Medicine, First Affiliated Hospital of Xi'an Jiaotong University, Xi'an, 710061, P.R. China.; 2 Department of Pathology, First Affiliated Hospital of Xi'an Jiaotong University, Xi'an, 710061, P.R. China.; 3 Department of Geriatric Surgery, First Affiliated Hospital of Xi'an Jiaotong University, Xi'an, P710061, P.R. China.; 4 Department of Epidemiology and Biostatistic School of Public Health & Global Health Institute, Xi'an Jiaotong University Health Science Center, Xi'an, 710061, P.R. China.

**Keywords:** Apoptosis-related genes, Prognostic model, Lung adenocarcinoma, Immunohistochemical, Dataset

## Abstract

The prognostic roles of apoptosis-related genes (ARGs) in lung adenocarcinoma (LUAD) have not been fully elucidated. In this study, differentially expressed genes (DEGs) associated with apoptosis and the hub genes were further identified. The prognostic values of the ARGs were evaluated using the LASSO Cox regression method. Prognostic values were determined using Kaplan-Meier (K-M) curves and receiver operating characteristic (ROC) curves in the TCGA and GEO datasets. The correlations, mutation data, and protein expression of the 10 ARGs predictive models were also analyzed. We identified 130 differentially expressed ARGs. DEGs were used to split LUAD cases into two subtypes whose overall survival (OS) were significantly different (*P* = 0.025). We developed a novel 10-gene signature using LASSO Cox regression. In both TCGA and GEO datasets, the results of the K-M curve and log-rank test showed significant difference in the survival rate of patients in the high-risk group and low-risk group (*P* < 0.0001). According to the GO and KEGG analyses, ARGs were enriched in cancer-related terms. In both cohorts, the immune status of the high-risk group was significantly lower than that of the low-risk group. Based on the differential expression of the ARGs, we established a new risk model to predict the prognosis of patients with LUAD.

## Introduction

Lung cancer is one of the most common malignancies that threaten human health. Over the past decade, the biology of lung cancer at the molecular level has improved, resulting in the development of effective therapies that improve overall survival 1, 2. Despite this decline in mortality, it is estimated that approximately 140,730 people will die of lung cancer by 2020 3. Approximately 85% of lung cancers are non-small cell lung cancers (NSCLC), with lung adenocarcinoma (LUAD) being the most prevalent type. LUAD shows distinct genetic drivers and divergent prognostic profiles compare to other types of lung cancer 4-6. Therefore, further research on the pathogenesis, development, and prognosis of LUAD is required to identify novel therapeutic targets.

Mammalian apoptosis, also known as programmed cell death, involves the removal of DNA-damaged cells, maintenance of tissue homeostasis, and regulation of cell growth 7-10. It may be triggered via way of means of pathways, inclusive of extrinsic and intrinsic pathways 11, while tumor cells can avoid apoptosis in several ways 12-14. Therefore, inducing cell apoptosis may be a strategy for tumor therapy 15, 16. Several studies have shown that apoptosis-related genes (ARGs) play a critical role in the treatment of lung cancer 17-20. In the current study, we determined the levels of ARGs in LUAD and normal lung specimens, examined the predictive significance of ARGs, and identified the correlations, mutational data, and tumor immune microenvironments that they were selected for further analysis. Our data also offer additional evidence for prognostic biomarkers and healing goals in LUAD.

## Materials and Methods

### Datasets and Sample Extraction

According to the TCGA database, we acquired 594 samples for RNA sequencing (RNA-seq) and clinical characteristics, including 535 LUAD samples and 59 normal samples (https://portal.gdc.cancer.gov/repository). RNA-seq data and clinical information for the external validation cohort, including 442 LUAD patient samples and related clinical features were collected from the GEO database (https://www.ncbi.nlm.nih.gov/geo ID: GSE68465). The inclusion criteria were: (1) patients diagnosed with LUAD and (2) patients with complete AGRs data and clinical information. The exclusion criteria were: (1) samples with incomplete clinical information or lack of AGRs related gene expression, and (2) follow-up time of less than 30 days.

### Identification of differentially expressed ARGs

A total of 580 ARGs were gained by the gene set “GOBP_APOPTOTIC_SIGNALING_PATHWAY” in the Molecular Signatures Database v7.1 in GSEA. Before comparison, expression data were normalized to fragments per kilobase of millions of values. The “limma” package was used to identify differentially expressed genes with a P value < 0.05 and |log FC| >1. The Search Tool for Retrieval of Interacting Genes (STRING) was used to generate protein-protein interaction (PPI) networks (https://string-db.org/).

### Development and validation of the ARGs prognostic model

Cox regression analysis was performed to determine the predictive value of ARGs and assess the correlation between each gene and survival status in TCGA cohort. The cut-off was set at P-value at 0.01 and 11 survival-related genes were identified. We then used the LASSO-Cox regression model (R package "glmnet") to narrow down candidate genes and broaden predictive models. Finally, 10 genes and their coefficients were confined, and the penalty parameter (λ) was determined by the minimum criterion. We calculated and created the risk score based on a linear combination of the ARGs formula multiplied by the regression coefficient (β): risk score =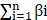
∗ (expression of 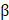
i). In the TCGA training cohort, patients with LUAD were divided into low- and high-risk subgroups based on the median risk score, and the OS times were compared using Kaplan-Meier analysis. PCA based on the 10-gene signature was performed by the “prcomp” function in the “stats” R package. And then the “survival” “survminer” and “time ROC” R packages were used to perform ROC curve analysis. The LUAD cohort from the GEO database (GSE68465) was used for the validation studies. Subsequently, we retrieved clinical data from patients in the TCGA and GEO cohorts. Univariate and multivariate Cox regression models were used for the analysis.

### Functional enrichment analysis between the low- and high-risk groups

The median risk score was used to separate patients with LUAD into two subgroups. DEGs were classified as low-risk or high-risk based on certain criteria (|log2FC| ≥ 1 and FDR < 0.05). We performed GO and KEGG analyses using the “clusterProfiler” package based on these DEGs. To calculate the scores of invading immune cells and the activity of immune-related pathways, the "gsva" package and the ssGSEA algorithm were employed.

### Cell Culture

The human LUAD cell lines (A549 and PC9) and normal human lung epithelial cell line (BEAS-2B) used in this study were cultured at 37°C in Dulbecco's modified Eagle's medium (11995065; Gibco, USA) and RPMI-1640 medium with 10% fetal bovine serum (FBS; Gemini Bio, USA) supplemented with 1% penicillin and streptomycin (15140122; Gibco). The cells were placed in a constant temperature incubator with a CO2 concentration of 5% and a temperature of 37°C.

### RNA Extraction and Real-Time PCR

Total RNA was extracted from cultured cells using TRIzol reagent (Invitrogen, USA), according to the manufacturer′s instructions. The concentration of the extracted RNA was controlled at 500 ng/ml, with purity between 1.80 and 2.00. Subsequently, RNA was reverse-transcribed into cDNA using PrimeScript™ RT Master Mix (RR036A, TaKaRa, Tokyo, Japan). The generated cDNA was amplified with primer pairs for the indicated gene to perform qRT-PCR on Applied Biosystems StepOnePlus Real-Time PCR System (ThermoFisher Scientific, MA, USA) by using TB Green® Premix Ex Taq™ II (RR820A, TaKaRa, Tokyo, Japan). Quantifications of target genes were normalized to relative levels of GADPH and expressed as ΔCt. The data was analyzed using the 2^-ΔΔCt^ method. Primers used for real-time PCR assays as follows: BCL2L10, Forward: 5′-GCTGGAGAGAGGGCCGCTGGTGA-3′ Reverse: 5′-TGGTGAAGACGCCAGTGGA-3′; MELK, Forward: 5′-TATTCACCTCGATGATGATTGCG-3′; Reverse: 5′-AGAAAGCCTTAAACGAACTGGTT-3′; ERO1A, Forward: 5′-ATCCTGAGCGCTACACTGGT-3′; Reverse: 5′-CTTGTCCCTTGACCAGAAGC-3′; KRT8, Forward: 5′-TCCTCAGGCAGCTATATGAAGAG-3′; Reverse: 5′-GGTTGGCAATATCCTCGTACTGT-3′; KRT18, Forward: 5′-GTTGACCGTGGAGGTAGATGC-3′; Reverse: 5′-GAGCCAGCTCGTCATATTGGG-3′; DDIT4, Forward: 5′-CTTTGGGACCGCTTCTCGTC-3′;Reverse: 5′-GGTAAGCCGTGTCTTCCTCCG-3′; PERP, Forward: 5′-CTTCACCCTTCATGCCAACC-3′; Reverse: 5′-GCCAATCAGGATAATCGTGGCT-3′; BTK, Forward: 5′-GCTCAAAAACGTAATCCGGTACA-3′; Reverse: 5′-GTCTTCCGGTGAGAACTCCC-3′; CX3CR1, Forward: 5′-ACTTTGAGTACGATGATTTGGCT-3′; Reverse: 5′-GGTAAATGTCGGTGACACTCTT-3′; DAPK2, Forward: 5′-CATCCTTGAGCTAGTGTCTGGA-3′; Reverse: 5′-GGATCTGCTTAATGAAGCTGGT-3′; GADPH, Forward: 5′-GAACGGGAAGCTCACTGGCATGGC-3′; Reverse: 5′-TGAGGTCCACCACCCTGTTGCTG-3.'

### 2.7 Protein extraction and western blot analysis

Total protein from the cell samples was lysed in cell lysis buffer for western blotting as previously described. Protein concentrations were quantified using a bicinchoninic acid kit (Beyotime, China). Proteins from each sample were separated by 10% SDS-PAGE and electroblotted onto polyvinylidene fluoride membranes (Millipore, Billerica, MA, USA). The membranes were blocked using 5% non-fat milk for 60 min at room temperature and incubated with primary antibodies overnight at 4°C while shaking. Primary antibodies against BCL2L10 (GeneTex, USA, 111872,1:1000 dilution), MELK (Proteintech, USA, 11403-1-AP, 1:1000 dilution), ERO1A (Proteintech, USA, 12007-1-AP, 1:1000 dilution), KRT8 (Proteintech, USA, 27105-1-AP, 1:1000 dilution), KRT18 (Proteintech, USA, 10830-1-AP,1:1000 dilution), DDIT4 (Proteintech, USA, 10638-1-AP, 1:1000 dilution), PERP (GeneTex, USA, 135223,1:1000 dilution), BTK (Proteintech, USA, 21581-1-AP, 1:1000 dilution), CX3CR1 (Proteintech, USA, 13885-1-AP, 1:1000 dilution), DAPK2 (Proteintech, USA, 20048-1-AP, 1:1000 dilution), and β-actin (ImmunoWay, TX, YM3028, 1:1000 dilution). The samples were then re-blotted with secondary antibodies (anti-mouse, Zhuangzhi Bio, China, EK010, 1:5000 dilution; anti-rabbit, Zhuangzhi Bio, China, EK020, 1:5000 dilution) at room temperature for 1.5h. Bioluminescence was detected using Image Lab software (Bio-Rad) and quantified using Image J software.

### Statistical analysis

Categorical variables were analyzed using the Pearson chi-square test, whereas gene expression levels in normal lung and LUAD tissues were compared using a single-factor analysis of variance. The Kaplan-Meier method was used to compare patient OS between subgroups using a two-sided log-rank test. The prognostic value of the risk model was evaluated using univariate and multivariate Cox regression analyses. Immunological cell infiltration and immune pathway activation were compared between the two groups using the Mann-Whitney test. All statistical analyses were performed using the R software (v 4.0.2).

## Results

### Identification of differentially expressed ARGs and classification in LUAD

**Figure 1:**
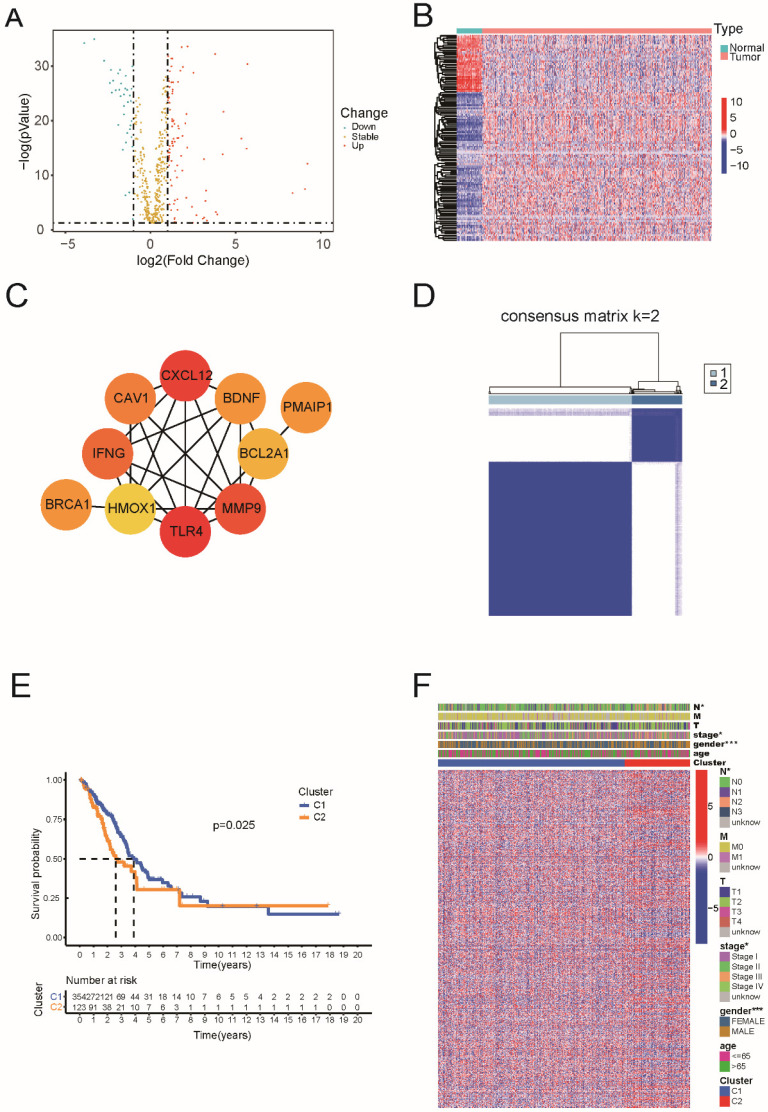
Identification of differentially expressed of 130 apoptosis-related genes and classification in LUAD. (A) The volcano plot and heatmap of apoptosis-related DEGs in LUAD. (B) The heatmap of apoptosis-related DEGs in LUAD. (C) The hub genes in the protein-protein interaction (PPI) analysis of apoptosis-related DEGs in LUAD. (D) 477 LUAD patients were grouped into two clusters according to the consensus clustering matrix (k=2). (E) Kaplan-Meier OS curves for the two clusters. (F) The heatmap and the clinic pathologic characters of the two clusters classified by these DEGs.

Based on TCGA data, which including 59 normal and 535 tumor tissues, we identified 130 genes of the 580 ARGs with differential expression (all P < 0.05 and |log2FC| ≥ 1). Among these 36 genes were downregulated and 94 genes were enriched within the LUAD group (Figure 1A and Supplementary Table 1). The RNA levels are presented as a heat map in Figure 1B. For PPI analysis, we used a minimum interaction score of 0.9, and we found that TLR4, CXCL12, MMP9, IFNG, CAV1, PMAIP1, BDNF, BRCA1, BCL2A1, and HMOX1 were hub genes (Figure 1C).

Next, we performed consensus clustering analysis on all 477 patients with LUAD to examine the relationship between the expression of 130 apoptosis-related DEGs and LUAD subtypes in TCGA dataset.

The inter-group correlation was low, and the intra-group correlation was strong as we increased the clustering variable (k) from 2 to 10 (k = 2), suggesting that LUAD patients may be effectively classified into two clusters based on these apoptosis-related DEGs (Figure 1D). According to Figure 1E, there was a statistically significant difference in overall survival between the two clusters (P = 0.025). The heatmap indicates the gene expression profile, together with clinical characteristics such as TNM stage, sex, and age (65 or >65 years). We found a substantial difference in the TNM stage and sex between the two groups (Figure 1F).

### 3.2 Development of a prognostic gene model in the TCGA cohort and validation in the GEO cohort

**Figure 2:**
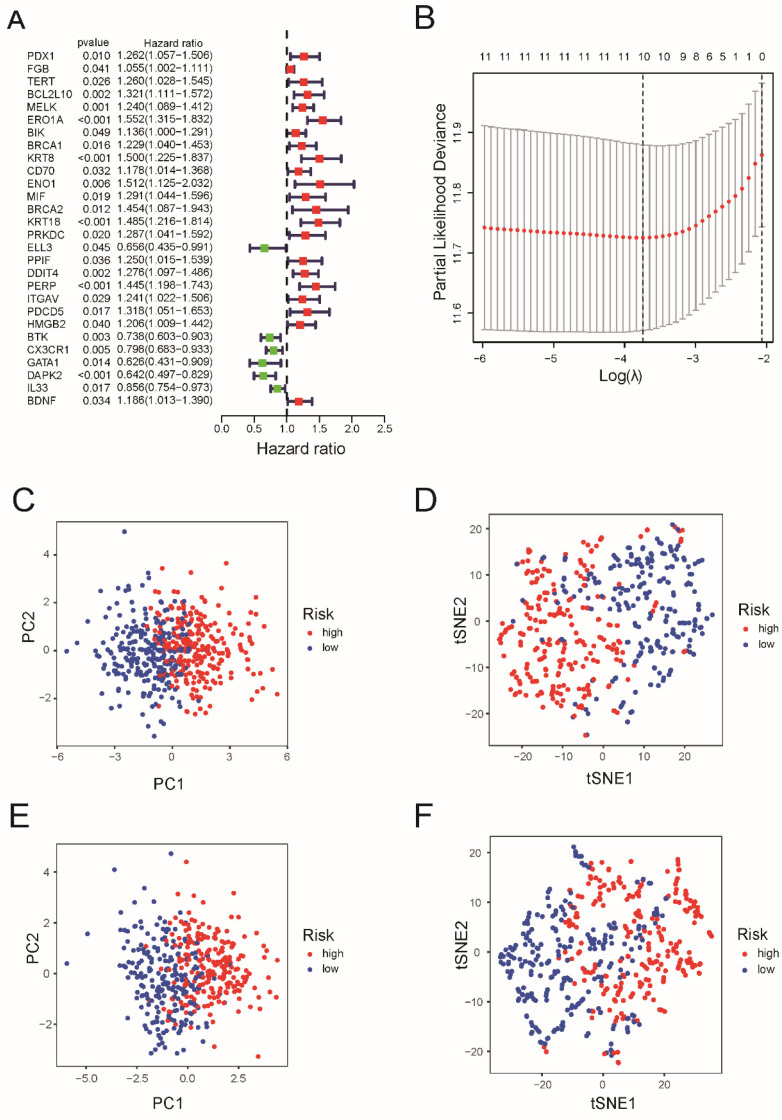
Construction of risk signature in the TCGA cohort and validation of the risk model in the GEO cohort. (A) Univariate cox regression analysis of OS for each apoptosis-related gene, and 28 genes with *P* < 0.05. (B) LASSO regression of the 7 OS-related genes. (C) Cross-validation for tuning the parameter selection in the LASSO regression. (D) PCA plot and (E) tee-snee for OCs based on the risk score in TCGA cohort. (F) PCA plot and (G) tee-snee for OCs based on the risk score in GEO cohort.

A total of 477 samples with complete survival information were matched to patients with LUAD. Univariate Cox regression analysis was used to conduct a preliminary search for genes associated with survival and the results showed that 28 genes (PDX1, FGB, TERT, BCL2L10, MELK, ERO1A, BIK, BRCA1, KRT8, CD70, ENO1, MIF, BRCA2, KRT18, PRKDC, ELL3, PPIF, DDIT4, PERP, ITGAV, PDCD5, HMGB2, BTK, CX3CR1, GATA1, DAPK2, IL33, and BDNF) satisfied the criteria of P < 0.05 (Figure 2A).

After LASSO Cox regression analysis, ten gene signatures (BCL2L10, MELK, ERO1A, KRT8, KRT18, DDIT4, PERP, BTK, CX3CR1, and DAPK2) were constructed based on the optimum λ value (Figure 2B). As shown in Supplementary Table 2, the risk score = (0.027 × BCL2L10 × exp.) + (0.016 × MELK × Exp) + (0.247*ERO1A exp.) + (0.033*KRT8 exp.) + (0.061*KRT18 exp.) + (0.110*DDIT4 exp.) + (0.097 × PERP × Exp) + (-0.113*BTK exp.) + (-0.048*CX3CR16 exp.) + (-0.039*DAPK2 exp.). Both PCA and tee-snee (t-SNE) both indicated that patients in the TCGA cohort were clearly divided into two clusters (Figure 2 C and 2D), as well as those in the GEO cohort (Figure 2E and 2F).

**Figure 3:**
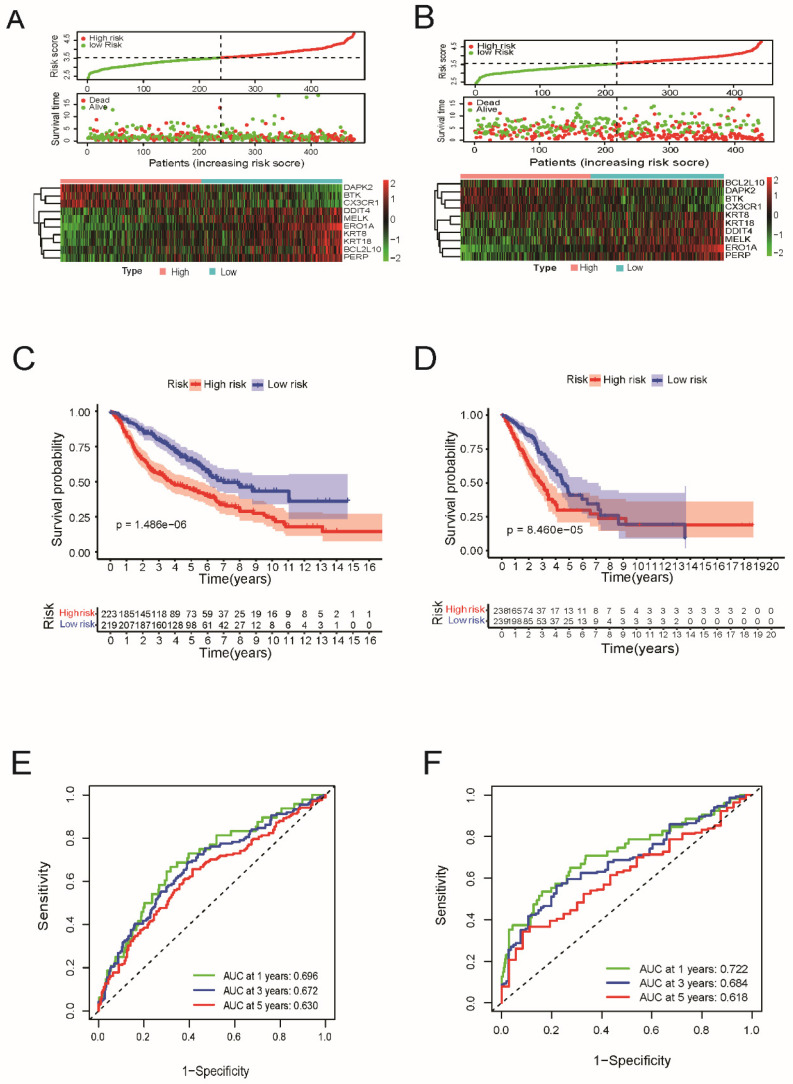
Prognostic value of the apoptosis-related gene signature in lung adenocarcinoma. (A) Risk score distribution, survival status and expression heatmap of 10 prognostic ARGs in LUAD patients in the TCGA cohort and (B) in the GEO cohort. (C) Kaplan-Meier overall survival curves analysis for the patients assigned to high-risk and low-risk group in TCGA dataset and (D) in GEO dataset. (E) Receiver operating characteristic (ROC) analysis of the accuracy for the ARGs signature-based risk score in the TCGA dataset and (F) in the GEO dataset.

Next, a risk formula was used to divide the patients into high- and low-risk groups using TCGA and GEO datasets. As shown in Figure 3A and 3B, patients with higher risk scores tended to have higher mortality, and patients with longer survival tended to have lower risk scores. The results of the log-rank and K-M curves showed that patients in the low-risk and high-risk groups had a significant difference in survival in the TCGA and GEO datasets, respectively (both P < 0.0001, Figure 3C and 3D). The ROC curve results suggested that this gene signature works well for predicting the survival of patients with LUAD.

For 1-year survival, 3-year survival, and 5-year survival, the areas under the ROC curve (AUC) of the TCGA dataset were 0.722, 0.684, and 0.618, respectively (Figure 3E). For the GEO cohort, the ROC curve analysis showed that this model had good predictive power (AUC=0.696 for 1-year survival, 0.672 for 3-year survival, and 0.630 for 5-year survival) (Figure 3F).

### Independent prognostic value of the risk model, mutation data and immunohistochemical analysis

**Figure 4:**
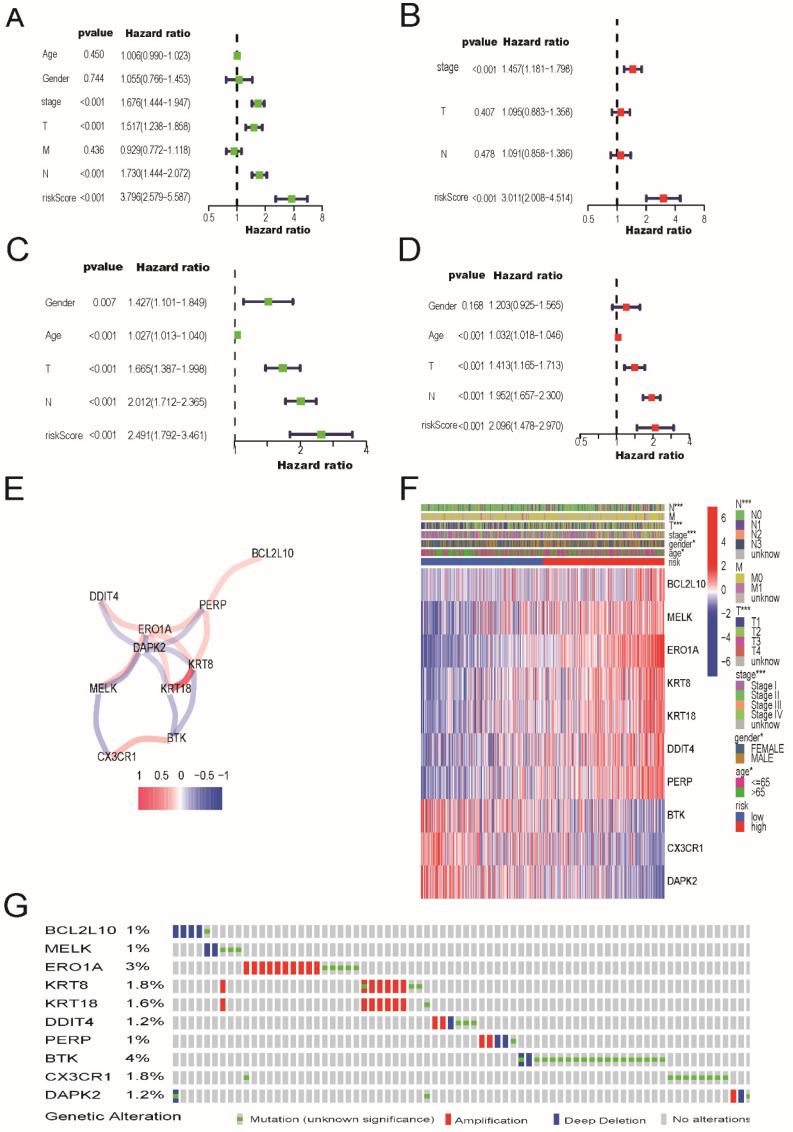
The univariate and multivariate Cox regression analyses, correlation network, clinic pathologic features, and mutation data of ARGs signature. (A) The univariate and (B) multivariate analysis for the TCGA cohort. (C) The univariate and (D) multivariate analysis for the GEO cohort. (E) The correlation network of the ARGs signature (red line: positive correlation; blue line: negative correlation. The depth of the colors reflects the strength of the relevance). (F) Heatmap (green: low expression; red: high expression) for the connections between clinic pathologic features and the risk groups (*P < 0.05, ***P < 0.001). (G) Mutation data of 10 screened ARGs in LUAD patients according to the cBioPortal database.

As shown in Figure 4A and 4C, in both the TCGA and GEO cohorts, univariate Cox regression analysis demonstrated that risk scores were independently associated with lower survival, as shown in Figure 4A and 4C (HR=3.796, 95% CI: 2.579-5.587 and HR: 2.491, 95% CI: 1.792-3.461, respectively). As shown in Figure 4B and 4D, multivariate analysis revealed that the risk score was a prognostic factor for patients with LUAD after accounting for other variables (HR=3.011, 95% CI, 2.008-4.514; HR, 2.096; 95% CI, 1.478-2.970, respectively). The correlation network containing the 10 genes in the signature model is shown in Figure 4E. As shown in Figure 4F, TNM stage, sex, and patient age were observed to be differentially distributed between the low-risk and high-risk categories when we created a heatmap of clinical features for the TCGA cohort (P < 0.05).

**Figure 5:**
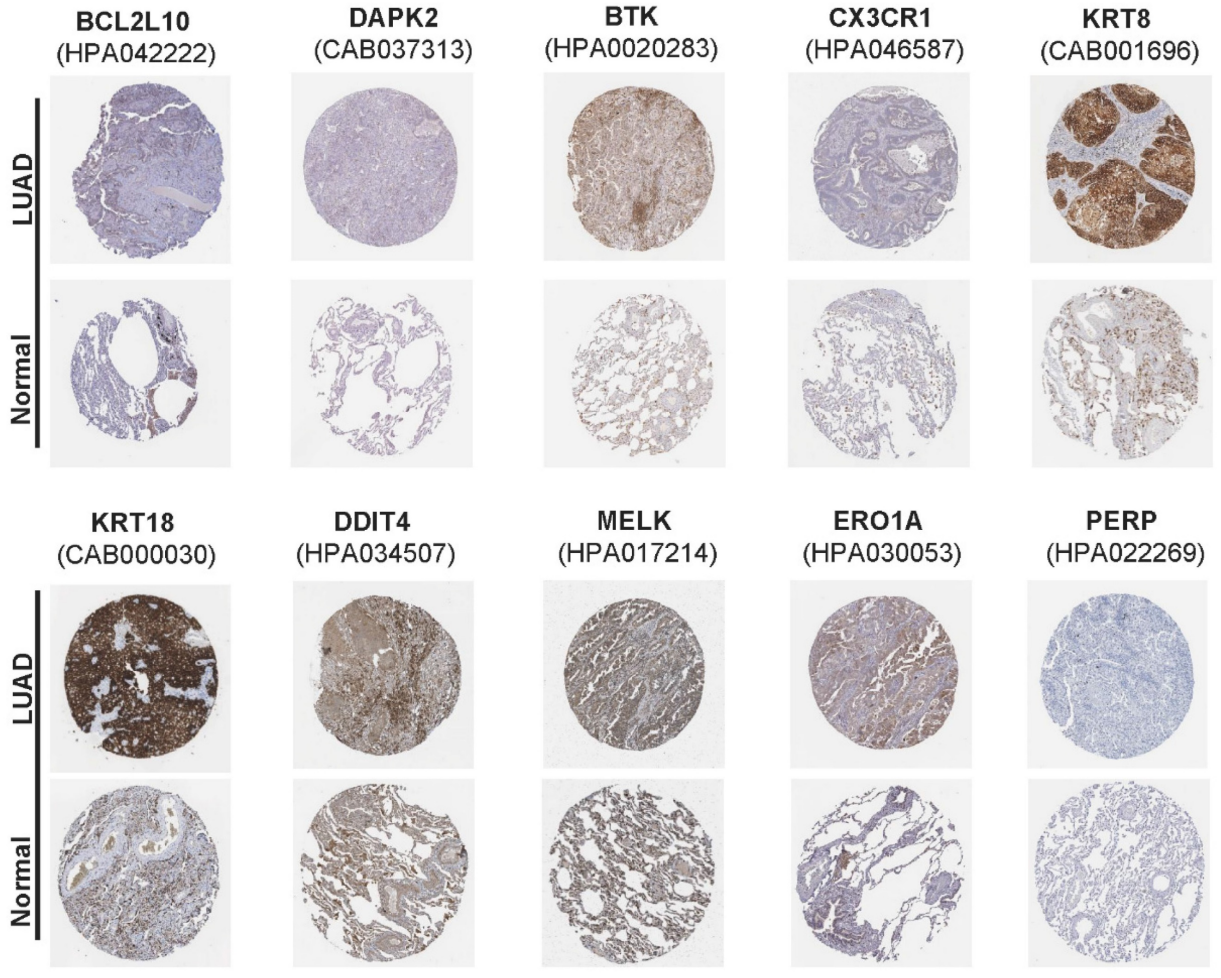
The immunohistochemical results of the expression levels of 10 screened ARGs between LUAD and para-carcinoma tissues according to the Human Protein Atlas (HPA) database.

In addition, mutation data for these 10 genes were analyzed using the cBioPortal database (http://cbioportal.org). A total of 507 patients with LUAD revealed that 75 (14.8%) had mutations. Among these 75 patients, 0.59% had deep deletions, 0.20% had mutations in BCL2L10, 0.39% had deep deletions, and 0.59% had mutations in MELK; 1.97% had amplifications, 0.79% had mutations in ERO1A; 1.38% had amplifications, 0.59% had mutations in KRT8; 1.38% had amplifications, 0.20% had mutations in KRT18; 0.39% had amplifications, and 0.20% had deep deletions, and 0.59% had mutations in DDIT4; and 0.39% had amplifications, 0.39% had deep deletions, and 0.20% had mutations in PERP. Among the protective genes in our model, 3.55% had mutations, 0.39% showed deep deletions in BTK, and 1.77% had mutations in CX3CR1. Meanwhile, 0.39% had deep deletions, 0.59% had mutations, and 0.20% had amplifications in DAPK2 (Figure 4G). Immunohistochemical analysis of these 10 ARGs in the prognostic model using the Human Protein Atlas database is shown in Figure 5, revealing that seven genes (BCL2L10, MELK, ERO1A, KRT8, KRT18, DDIT4, and PERP) were significantly upregulated in LUAD tissues.

### Functional and immune activity analyses between subgroups of the risk model

**Figure 6:**
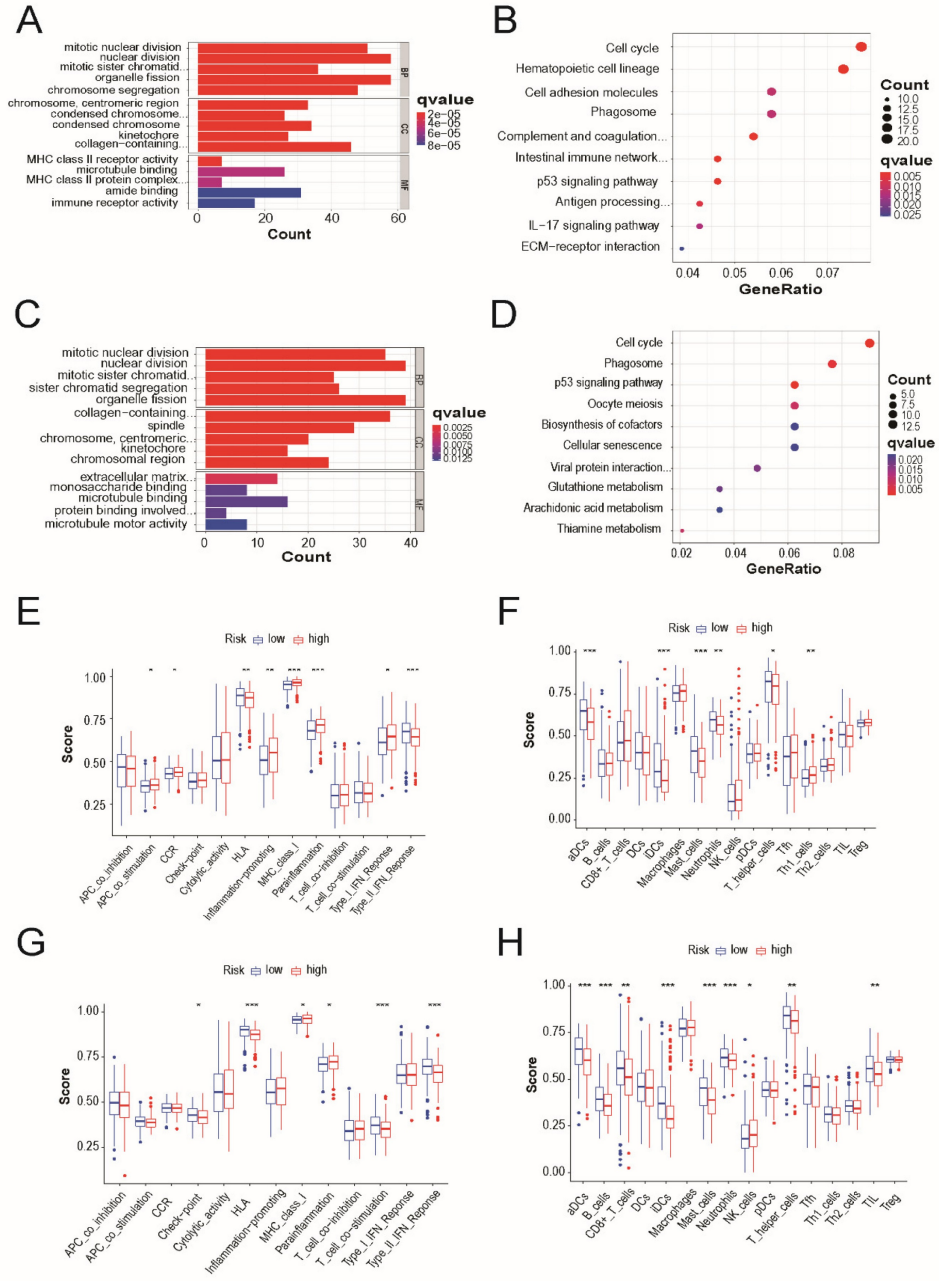
Functional and immune activity analyses between subgroups of the risk model. (A) Barplot graph for GO enrichment and (B) Bubble graph for KEGG pathways in TCGA cohort. (C) Barplot graph for GO enrichment and (D) Bubble graph for KEGG pathways in GEO cohort. The bigger bubble means the more genes enriched, and the increasing depth of red means the differences were more obvious; q-value: the adjusted p-value; the longer bar means the more genes enriched, and the increasing depth of red means the differences were more obvious. Comparison of the enrichment scores of (E) 16 types of immune cells and (F) 13 immune-related pathways between low- (green box) and high-risk (red box) group in the TCGA cohort. Comparison of the enrichment scores of (G) 16 types of immune cells and cells and (H) 13 immune-related pathways between low- (blue box) and high-risk (red box) group in the GEO cohort. *P* values were showed as: *P < 0.05; **P < 0.01; ***P < 0.001.

We identified 90 DEGs between the low- and high-risk groups in the TCGA cohort. In the high-risk group, 39 genes were upregulated and 51 genes were downregulated. Gene Ontology and Kyoto Encyclopedia of these DEGs, GO and Genomes pathway analyses were performed based out. The results demonstrated that cell cycle, p53 signaling pathway, and phagosomes were specifically associated with these DEGs (Figure 6A and 6B). Similar results were observed in the external validation of the GEO cohort (Figure 6C and 6D).

The accumulation levels of 16 different immune cell types and the activities of 13 immune-related pathways were then compared across the low- and high-risk groups in both TCGA and GEO cohorts using ssGSEA. In TCGA cohort, high-risk subgroups had more common infiltration levels of immune cells, particularly activated dendritic cells, immature dendritic cells, master cells, neutrophils, and T helper cells (Figure 6E). In TCGA cohort, the HLA and type-2 IFN response pathways showed lower reactivity in the high-risk group than in the low-risk group. (Figure 6F). Similar outcomes were observed when examining the immunological state and pathways in the GEO cohort (Figure 6G and 6H).

### 3.5 The analysis of qRT-PCR and Western blot for AGRs genes in lung cancer cell lines

**Figure 7:**
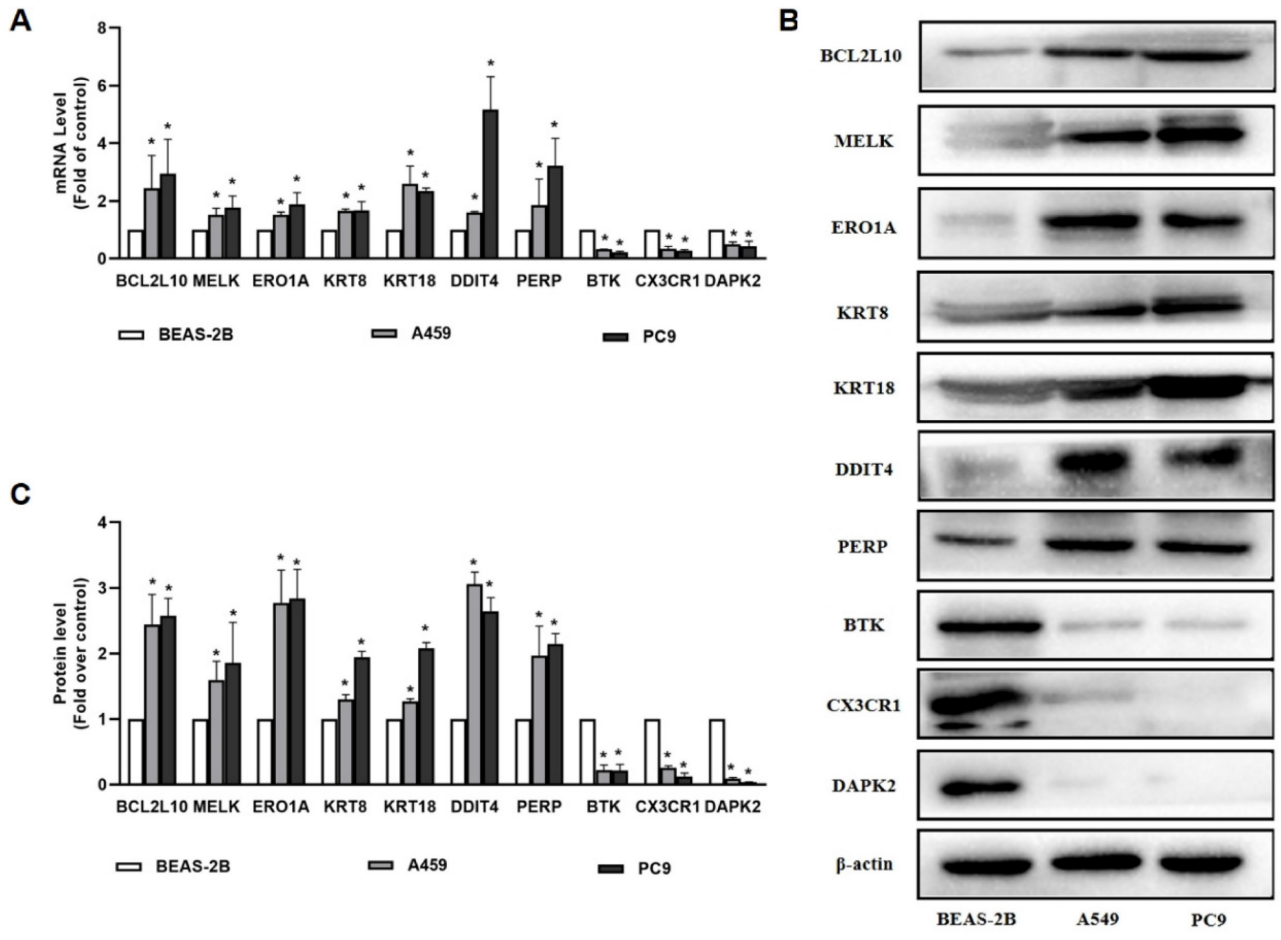
The results of qRT-PCR and Western blot for 10 ARGs genes. (A) The expression of BCL2L10, MELK, ERO1A, KRT8, KRT18, DDIT4, PERP, BTK, CX3CR1 and DAPK2 determined by qRT-PCR in A549 and PC9 cells; (B-C) The expression of BCL2L10, MELK, ERO1A, KRT8, KRT18, DDIT4, PERP, BTK, CX3CR1 and DAPK2 determined by Western blot in A549 and PC9 cells. *P* values were showed as: *P < 0.05.

In our study, 10 apoptosis-related genes were identified using TCGA and GEO datasets, which are essential for the pathogenesis of LUAD. Among these genes, MELK, ERO1A, and KRT18 were well established by reports that they were overexpressed in patients with LUAD compared to healthy patients. Consequently, BCL2L10, MELK, ERO1A, KRT8, KRT18, DDIT4, PERP, BTK, CX3CR1, and DAPK2 were selected as the genes of interest and were validated by qRT-PCR and western blotting. As shown in Figure 7, our results demonstrates that BTK, CX3CR1, and DAPK2 were expressed at lower levels in A549 and PC9 cells than in normal bronchial epithelial cells. BCL2L10, MELK, ERO1A, KRT8, KRT18, DDIT4, and PERP levels were higher in A549 and PC9 cells than in normal bronchial epithelial cells, which was consistent with our predictions (Figure 7).

## Discussion

In the present study, we analyzed the expression of 580 ARGs in LUAD and normal tissues, of which 130 were DEGs. Importantly, the two clusters produced by consensus clustering analysis based on apoptosis-associated DEGs showed extensive variation in clinical characteristics. We constructed a 10-generisk signature (BCL2L10, MELK, ERO1A, KRT8, KRT18, DDIT4, PERP, BTK, CX3CR1, and DAPK2) using Cox univariate and LASSO Cox regression analyses. This apoptosis-related prognostic risk score model was established to perform well on an external dataset. We also found that approximately 15% of the patients with LUAD had gene mutations. Following functional studies, we discovered that the primary associations between the DEGs in the low-risk and high-risk groups were the cell cycle, p53 signaling pathway, and phagosome pathways. Finally, our findings showed that, compared to the low-risk group, the high-risk group had significantly fewer invading immune cells and lower activity of immune-related pathways.

Apoptotic pathways play a critical role in resistance to conventional anticancer therapies, including radiotherapy, chemotherapy, and targeted therapy 21. We generated a signature featuring 10 apoptosis-related genes and found that it could predict the OS of patients with LUAD. Most genes in our signature were involved in cancer, including lung cancer. For example, BCL2L10 was found to be overexpressed in breast, prostate, colorectal, and lung cancers, as well as in multiple myeloma, and increased BCL2L10 expression was correlated with poor prognosis 22-25. Recently, Tang *et al.* demonstrated that MELK plays an important role in metastasis, mitotic progression, and programmed cell death in LUAD 26. ERO1A, which plays a key role in protein synthesis, is an oncogenic promoter of NSCLC and may promote NSCLC development by regulating cell cycle-associated molecules, such as cyclin D1 and CDK6 27. In this study, we also found that the ERO1A gene was upregulated in LUAD patients from the TCGA and GEO datasets, and its amplification was common. A recent study suggested that patients with high KRT8 expression had significantly reduced overall survival and recurrence-free survival 28. Wang *et al.* demonstrated that KRT8 is hypomethylated, overexpressed, and associated with poor prognosis in LUAD 29. Patients with NSCLC with high KRT18 expression had poorer OS and DFS than those with low KRT18 expression. KRT18 knockdown reduced cell migration and promoted paclitaxel-induced apoptosis in lung cancer cells 30.

A previous study reported that DDIT4 higher expression correlated with lower overall survival in patients with LUAD 31, which is consistent with our results. PERP was first identified as an effector of p53, which is involved in apoptosis. Liao *et al.* demonstrated that PERP is highly expressed in lung cancers, as well as in the TCGA cohort 32, and further revealed that ectopic expression of PERP-428G protects lung cancer cells from ROS-induced DNA damage. BTK has been found to negatively correlate with clinicopathological characteristics and positively correlate with the overall survival of patients with LUAD 33. In TCGA and GSE68465 datasets, we found that CX3CR1 expression was significantly lower than normal in LUAD patients, and high CX3CR1 expression was associated with improved survival outcomes in LUAD. According to a recent study, the serum levels of CX3CL1 and CX3CR1 in the bone metastasis group were considerably higher than those in the bone metastasis and healthy control groups 34. Jin *et al.* found that downregulation of DAPK2 promotes NSCLC cell proliferation and migration in vitro and in vivo through activating NF-κB signaling pathway 35. As we observed that 10 ARGs have a potential cancer-promoting role in LUAD, we performed further experiments in lung cancer cell lines to validate their carcinogenic role. We found that BCL2L10, MELK, ERO1A, KRT8, KRT18, DDIT4, and PERP levels were higher in A549 and PC9 cells. We also determined that the expression of BTK, CX3CR1, and DAPK2 was lower in A549 and PC9 cells than in normal bronchial epithelial cells, which is consistent with database predictions. Taken together, the functional roles of these 10 genes in the LUAD signature may provide a reference for further research.

In conclusion, a novel 10-gene signature model based on ARGs in LUAD was developed and validated. This model can be used to predict LUAD outcomes. However, well-designed prospective trials are needed to further evaluate its predictive significance and confirm its clinical utility.

## Supplementary Material

Supplementary tables.

## Figures and Tables

**Table 1:** The 130 apoptosis-related DEGs of LUAD.

Type	Genes
Up-regulated	PDX1|FGB|MAGEA3|TERT|POU4F1|RNF186|BCL2L10|RNF183|MAEL|AVP|MELK|SCG2|AGT|IL19|ERN2|GDNF|RET|FZD9|E2F2|SCRT2|IFNB1|ERO1A|MMP9|BIK|BRSK2|TNFRSF25|PDK1|ATP2A1|MLLT11|GGCT|PMAIP1|FIGNL1|DEPTOR|E2F1|BRCA1|CHEK2|MAPK8IP2|SCN2A|NOX1|CD24|BCL2L14|FGG|CD27|SFRP2|TRIB3|EPO|KRT8|IFNG|GATA4|CD70|NFATC4|ITM2C|ENO1|MIF|BRCA2|POLB|IKBKE|TRAP1|HYOU1|CTH|KRT18|PRKDC|BNIP3|ELL3|ASAH2|PPIF|DDIT4|P4HB|PDCD6|INHBB|GCLM|TRAF2|SGPL1|PERP|PIDD1|SSTR3|BCL2L12|DAP|TMEM117|ITGAV|PARP1|SFN|BMF|MSH2|SLC9A3R1|CHAC1|NLE1|PDIA3|PDCD5|DAP3|DNAJC10|NOL3|HMGB2|DYRK2|
Down-regulated	MIR222|BCL2A1|TNFSF12|CXCL12|PTGIS|PPM1F|HYAL2|CASP5|EYA4|BTK|MIR221|TYROBP|PAK5|TLR4|CX3CR1|SRPX|HMOX1|ITPRIP|ZNF385B|GATA1|IL20RA|ATF3|PPP1R15A|TIMP3|DAPK2|LRRK2|PF4|IL33|GPER1|BDNF|AGTR2|CASP12|DCC|FGF10|CAV1|RTKN2|

DEGs, differentially expressed genes; LUAD, lung adenocarcinoma

**Table 2:** LASSO regression coefficients of ten ARGs in LUAD.

Gene ID	Coefficient	HR	HR.95L	HR.95H	*P* value
BCL2L10	0.026925765	1.321110306	1.110507276	1.571653315	0.001672372
BTK	-0.113009996	1.136421628	1.000362187	1.290986538	0.049352969
CX3CR1	-0.048166906	0.798012156	0.682597644	0.932941107	0.004642533
DAPK2	-0.03935147	0.641598542	0.4966291	0.828885559	0.000683431
DDIT4	0.110288702	1.276489596	1.096783002	1.485640902	0.001614492
ERO1A	0.247294298	1.552152017	1.315108979	1.831921097	2.00E-07
KRT18	0.061318726	1.484738161	1.21553171	1.81356635	0.000107873
KRT8	0.033458491	1.499757744	1.224652087	1.836663092	8.85E-05
MELK	0.016112473	1.23966466	1.08853765	1.411773373	0.001199757
PERP	0.097117364	1.445216955	1.198028172	1.743408123	0.000119195

ARGs, apoptosis-related genes; LUAD, lung adenocarcinoma
