# Supplementary material for: Identification and validation of a novel apoptosis-related prognostic risk score model for lung adenocarcinoma
Source: J Cancer. 2024 Apr 28;15(11):3381–93. doi: 10.7150/jca.92616 (PMC11134425; doi:10.7150/jca.92616)
Supplement: Supplementary file 1 — Supplementary tables. [file jcav15p3381s1.zip › Supplementary table 2.docx]

**Table 2** LASSO regression coefficients of ten ARGs in LUAD.

| **Gene ID** | **Coefficient** | **HR** | **HR.95L** | **HR.95H** | ***P* value** |
| --- | --- | --- | --- | --- | --- |
| BCL2L10 | 0.026925765 | 1.321110306 | 1.110507276 | 1.571653315 | 0.001672372 |
| BTK | -0.113009996 | 1.136421628 | 1.000362187 | 1.290986538 | 0.049352969 |
| CX3CR1 | -0.048166906 | 0.798012156 | 0.682597644 | 0.932941107 | 0.004642533 |
| DAPK2 | -0.03935147 | 0.641598542 | 0.4966291 | 0.828885559 | 0.000683431 |
| DDIT4 | 0.110288702 | 1.276489596 | 1.096783002 | 1.485640902 | 0.001614492 |
| ERO1A | 0.247294298 | 1.552152017 | 1.315108979 | 1.831921097 | 2.00E-07 |
| KRT18 | 0.061318726 | 1.484738161 | 1.21553171 | 1.81356635 | 0.000107873 |
| KRT8 | 0.033458491 | 1.499757744 | 1.224652087 | 1.836663092 | 8.85E-05 |
| MELK | 0.016112473 | 1.23966466 | 1.08853765 | 1.411773373 | 0.001199757 |
| PERP | 0.097117364 | 1.445216955 | 1.198028172 | 1.743408123 | 0.000119195 |

ARGs, apoptosis-related genes; LUAD, lung adenocarcinoma

**Table 3** Clinical influences of risk score signature for TCGA-LUAD data.

| Clinical | n | Risk Score | | *p* |
| --- | --- | --- | --- | --- |
|  |  | Mean | SD |  |
| Age |  |  |  | *P*=0.057 |
| ≤ 65 | 219 | 3.602 | 0.4394 |  |
| ＞65 | 238 | 3.523 | 0.4438 |  |
| Gender |  |  |  | *P*=0.0218 |
| Female | 243 | 3.518 | 0.4465 |  |
| Male | 214 | 3.613 | 0.4320 |  |
| Stage |  |  |  | *P*＜0.0001 |
| I-II | 358 | 3.514 | 0.4188 |  |
| III-IV | 99 | 3.740 | 0.4790 |  |
| T |  |  |  | *P*=0.0031 |
| T_1-2_ | 398 | 3.539 | 0.4334 |  |
| T_3-4_ | 59 | 3.721 | 0.4689 |  |
| M |  |  |  | *P*=0.3336 |
| M_0_ | 304 | 3.576 | 0.4513 |  |
| M_1_ | 153 | 3.534 | 0.4238 |  |
| N |  |  |  | *P*＜0.0001 |
| N_0_ | 301 | 3.484 | 0.4085 |  |
| N_1-2_ | 156 | 3.713 | 0.4666 |  |
