# Supplementary material for: Identification and validation of a novel apoptosis-related prognostic risk score model for lung adenocarcinoma
Source: J Cancer. 2024 Apr 28;15(11):3381–93. doi: 10.7150/jca.92616 (PMC11134425; doi:10.7150/jca.92616)
Supplement: Supplementary file 1 — Supplementary tables. [file jcav15p3381s1.zip › Supplementary table 1.docx]

**Table 1** The 130 apoptosis-related DEGs of LUAD.

| **Type** | **Genes** |
| --- | --- |
| Up-regulated | PDX1**\|**FGB**\|**MAGEA3**\|**TERT**\|**POU4F1**\|**RNF186**\|**BCL2L10**\|**RNF183**\|**MAEL**\|**AVP**\|**MELK**\|**SCG2**\|**AGT**\|**IL19**\|**ERN2**\|**GDNF**\|**RET**\|**FZD9**\|**E2F2**\|**SCRT2**\|**IFNB1**\|**ERO1A**\|**MMP9**\|**BIK**\|**BRSK2**\|**TNFRSF25**\|**PDK1**\|**ATP2A1**\|**MLLT11**\|**GGCT**\|**PMAIP1**\|**FIGNL1**\|**DEPTOR**\|**E2F1**\|**BRCA1**\|**CHEK2**\|**MAPK8IP2**\|**SCN2A**\|**NOX1**\|**CD24**\|**BCL2L14**\|**FGG**\|**CD27**\|**SFRP2**\|**TRIB3**\|**EPO**\|**KRT8**\|**IFNG**\|**GATA4**\|**CD70**\|**NFATC4**\|**ITM2C**\|**ENO1**\|**MIF**\|**BRCA2**\|**POLB**\|**IKBKE**\|**TRAP1**\|**HYOU1**\|**CTH**\|**KRT18**\|**PRKDC**\|**BNIP3**\|**ELL3**\|**ASAH2**\|**PPIF**\|**DDIT4**\|**P4HB**\|**PDCD6**\|**INHBB**\|**GCLM**\|**TRAF2**\|**SGPL1**\|**PERP**\|**PIDD1**\|**SSTR3**\|**BCL2L12**\|**DAP**\|**TMEM117**\|**ITGAV**\|**PARP1**\|**SFN**\|**BMF**\|**MSH2**\|**SLC9A3R1**\|**CHAC1**\|**NLE1**\|**PDIA3**\|**PDCD5**\|**DAP3**\|**DNAJC10**\|**NOL3**\|**HMGB2**\|**DYRK2**\|** |
| Down-regulated | MIR222**\|**BCL2A1**\|**TNFSF12**\|**CXCL12**\|**PTGIS**\|**PPM1F**\|**HYAL2**\|**CASP5**\|**EYA4**\|**BTK**\|**MIR221**\|**TYROBP**\|**PAK5**\|**TLR4**\|**CX3CR1**\|**SRPX**\|**HMOX1**\|**ITPRIP**\|**ZNF385B**\|**GATA1**\|**IL20RA**\|**ATF3**\|**PPP1R15A**\|**TIMP3**\|**DAPK2**\|**LRRK2**\|**PF4**\|**IL33**\|**GPER1**\|**BDNF**\|**AGTR2**\|**CASP12**\|**DCC**\|**FGF10**\|**CAV1**\|**RTKN2**\|** |

DEGs, differentially expressed genes; LUAD, lung adenocarcinoma
