# Supplementary material for: Identification and validation of a novel apoptosis-related prognostic risk score model for lung adenocarcinoma
Source: J Cancer. 2024 Apr 28;15(11):3381–93. doi: 10.7150/jca.92616 (PMC11134425; doi:10.7150/jca.92616)
Supplement: Supplementary file 1 — Supplementary tables. [file jcav15p3381s1.zip › Supplementary table 3.doc]

**Table 3** Clinical influences of risk score signature for TCGA-LUAD data.

| Clinical | n | Risk Score | | *p* |
| --- | --- | --- | --- | --- |
|  |  | Mean | SD |  |
| Age |  |  |  | *P*=0.057 |
| ≤ 65 | 219 | 3.602 | 0.4394 |  |
| ＞65 | 238 | 3.523 | 0.4438 |  |
| Gender |  |  |  | *P*=0.0218 |
| Female | 243 | 3.518 | 0.4465 |  |
| Male | 214 | 3.613 | 0.4320 |  |
| Stage |  |  |  | *P*＜0.0001 |
| I-II | 358 | 3.514 | 0.4188 |  |
| III-IV | 99 | 3.740 | 0.4790 |  |
| T |  |  |  | *P*=0.0031 |
| T_1-2_ | 398 | 3.539 | 0.4334 |  |
| T_3-4_ | 59 | 3.721 | 0.4689 |  |
| M |  |  |  | *P*=0.3336 |
| M_0_ | 304 | 3.576 | 0.4513 |  |
| M_1_ | 153 | 3.534 | 0.4238 |  |
| N |  |  |  | *P*＜0.0001 |
| N_0_ | 301 | 3.484 | 0.4085 |  |
| N_1-2_ | 156 | 3.713 | 0.4666 |  |
